# Impact of reinforcement additives on physical and electrical properties of fly ash-based geopolymer materials

**DOI:** 10.1038/s41598-026-46494-x

**Published:** 2026-04-13

**Authors:** Khadiga M. Abas, Rehab E. A. Ngida, Somia M. Abbas

**Affiliations:** 1 Physical Chemistry Department, Advanced Materials Technology and Mineral Resources Research Institute, National Research Centre, 33 El-Bohouth St., Giza, 12622 Egypt; 2 Refractories, Ceramics and Building Materials Department, National Research Centre, Dokki, Cairo, 12622 Egypt; 3 Inorganic Chemistry Department, National Research Centre, 33 El Bohouth St., (Former El Tahrir St.), Dokki, Giza 12622 Egypt

**Keywords:** Fly ash, Active quartz, Microcrystalline cellulose, Carbon fibers, Green geopolymers, Dielectric properties, Engineering, Environmental sciences, Materials science

## Abstract

The utilization of carbon-based additives, generated from waste managed materials, to synthesize fly ash (FA)-based geopolymers with enhanced mechanical and electrical properties offers benefits in environmental protection and waste reduction. This study focused on preparing FA-based geopolymers at ambient conditions through alkali activation with a combination of NaOH-activated quartz (AQ) and water glass (Na_2_SiO_3_ solution). The weight ratio of FA:AQ in the FA/AQ geopolymer was kept at 1:1 (wt:wt). Carbon-based additives, including carbon fibers (CFs) and thermally stabilized microcrystalline cellulose (SMCC), were separately mixed to FA/AQ geopolymer paste in two proportions (1% and 3% wt/wt) relative to FA. The formulated geopolymers were analyzed physically using Fourier transform infrared spectroscopy (FTIR), UV/Vis spectroscopy, X-ray diffraction (XRD), scanning electron microscopy (SEM), and EDX. SEM analysis reveals the presence of voids and cavities in the neat FA geopolymer. However, integrating AQ into the FA-based geopolymer leads to significant matrix densification and reduced porosity, restricting ion mobility, resulting in high mechanical strength, and low electrical conductivity. Additionally, the enhanced compatibility of a higher percentage of CFs and SMCC (CFs(3%)@FA/AQ and SMCC(3%)@FA/AQ) with the geopolymer matrix forms dense, amorphous sodium aluminum silicate hydrate (N-A-S–H) links. This is confirmed by the increased compressive strength (11.1 MPa and 18.1 MPa) and higher intensities of SMCC’s XRD patterns. SMCC(3%)@FA/AQ demonstrates the lowest electric and dielectric properties (σ = 1.4 × 10^–7^ S/cm and ε′ = 7 × 10^4^), indicating superior insulating properties. In contrast, the CFs(3%)@FA geopolymer matrix exhibits higher values (σ = 4.4 × 10^–6^ S/cm and ε′ = 2.5 × 10^6^) compared to other matrices after shielding AQ that interrupt the conductive pathways.

## Introduction

The construction materials domain is particularly noteworthy as it ranks among the most polluting industries. According to a 2019 report from the World Green Building Council, buildings and the construction industry contribute 39% of global carbon dioxide emissions ^1^. Therefore, partially or fully substituting traditional materials with those derived from sustainable sources and processed at lower temperatures would reduce gas emissions into the atmosphere ^2^. Geopolymer concrete is a newly emerging type of binder for concrete mixtures. This material serves as an alternative to traditional cement, featuring environmentally sustainable qualities, including the use of waste materials and reduced greenhouse gas emissions during production ^3,4^.

With objectives centered on sustainability, economy, and durability, the concept of geopolymers has progressed ^5^. Geopolymers are aluminosilicate materials ^6,7^ that exhibit amorphous or semi-crystalline structures, comprising AlO_4_ and SiO_4_ units that form a three-dimensional microstructure through polycondensation ^8,9^. They can be produced by activating aluminosilicate minerals and industrial by-products with alkali. The alkaline activation process typically employs sodium hydroxide (NaOH) and sodium silicate (Na₂SiO₃). The combination of hydroxyl ions from these activators causes amorphous silicon and aluminum to dissolve from the starting material, leading to the formation of a strong, three-dimensional geopolymeric structure through polycondensation ^3^. Over the past few decades, the materials used to make geopolymers have expanded beyond traditional kaolinite to include a variety of industrial byproducts rich in aluminosilicates, such as fly ash, red mud, natural and artificial pozzolans, blast furnace slag, and mine tailings ^10–12^. Fly ash, an industrial waste product generated from burning coal in power plants, is primarily composed of aluminum oxide (Al_2_O_3_) and silicon dioxide (SiO_2_) ^13^. Primarily utilized in construction, agriculture, chemistry, industry, and ceramics, fly ash presents significant storage and pollution challenges. Consequently, there is an increasing focus on fly ash recycling ^14,15^. Its potential as a sustainable material in concrete has been explored in several studies ^16–18^. Edvardsen et al. ^19^ contributed to this field by developing a fly ash-based concrete that minimizes CO_2_ emissions. They suggested using stainless steel reinforcement and a specialized cladding system to improve the durability of concrete structures. Wang et al. ^20^ explored the micromechanical properties of high-volume fly ash engineered cementitious composite (ECC). Their research indicated that incorporating fly ash enhances tensile strain capacity, but the optimal amount depends on the desired compressive strength. In recent years, geopolymers have gained attention as matrix materials for composite systems, enabling the creation of multifunctional materials. Beyond their conventional use in construction, these materials have found applications in energy-related areas ^21^, such as electrical conductivity, electromagnetic field shielding, electrostatic discharge prevention, and cathodic protection of reinforced concrete structures ^2^. Notably, the electrical conductivity of geopolymer matrices is strongly linked to percolation phenomena and the dispersion of reinforcing fillers ^22^. Carbon-based fillers can produce ionic and electronic conductivity in cementitious materials and enhance their compressive strength ^2,23^. These reinforcing materials may include particulate ^24^, short fibers, or continuous fibers. According to Vlachakis et al. ^25^, several alkaline-activated materials, particularly fly ash with NaOH, exhibit conductivity when combined with carbonaceous fillers like graphene and graphene oxide ^26^. Graphene and its derivatives ^27^ represent promising materials for enhancing conductivity due to their extensive π electron system, which facilitates ion transport. Zhong et al. ^28^ reported that the integration of reduced graphene oxide into geopolymer yields a ceramic composite with a high electrical conductivity of 1.8 × 10^2^ S/m. The authors also proposed improved mechanical properties due to the strong interfacial interaction between the geopolymer and graphene oxide. Short fibers are most suitable for civil engineering applications, considering cost efficiency, ease of handling, and manufacturing ^29^. Many studies ^30–32^ have explored composites integrated with various carbon-based fibers, such as polyvinyl alcohol (PVA), polyvinyl chloride (PVC), polypropylene (PP), carbon nanotubes (CNTs), and carbon fibers (CFs). Typically, CFs and CNTs are preferred over other carbon-based materials for electrical enhancement due to their lower electrical resistivity at high frequencies. Saafi et al. ^33^ introduced multi-walled carbon nanotubes (MWCNT) into the geopolymer matrix, stating that the conductivity of the MWCNT/geopolymer composite improved, reaching its highest value at 1 wt% MWCNT. Despite the potential of CNTs to improve the mechanical and electrical properties of cement composites, significant challenges remain. A key obstacle is achieving uniform dispersion of individual CNTs within the cement matrix ^34,35^. Due to their high aspect ratio and strong van der Waals forces, CNTs tend to agglomerate into bundles, hindering effective integration ^34,35^. While sonication with a water-soluble surfactant is frequently employed to improve the dispersion of CNTs in cement matrices ^36,37^, this method has a significant drawback. The surfactant can introduce air pores into the cement, ultimately weakening its mechanical properties ^37^. This limitation makes CFs a more attractive option than CNTs for industrial applications. CFs exhibit weaker van der Waals attraction between individual fibers, eliminating the need for surface modification. This saves costs and time while yielding high-quality final products.

Numerous studies have demonstrated the benefits of incorporating natural fibers, including micro and nano-cellulose, to enhance mechanical performance and reduce energy consumption during the manufacturing of building materials ^38–40^. The application of microcrystalline cellulose (MCC) in geopolymer technology is not extensively documented, and its use in Portland cement composites is primarily limited to mortars and pastes. MCC can interact with cement hydration products more intensely than cellulose pulps, potentially delaying the curing process, which boosts hydration and enhances mechanical behavior. Furthermore, it can help mitigate cracks caused by thermal contractions and structural restraints ^38^. Heating MCC causes substantial changes to its crystalline structure, thermal resistance, and chemical composition. When heated to a specific temperature (for example, 200 °C), the crystallinity of MCC can increase as its amorphous regions break down. This process also leads to decomposition, the development of both polar and non-polar components, and the creation of carbon-based materials. Research on the application of thermally-treated MCC and the interactions between geopolymer composites and lignocellulosic reinforcements is currently limited, highlighting a gap in its understanding ^41,42^.

Quartz is a chemically inactive silicate mineral that does not contribute to the geopolymerization process. In fact, it can weaken the geopolymer structure, ultimately reducing its compressive strength ^43^. To address this, alkali fusion can enhance quartz’s reactivity by destroying its crystal structure ^44^. Tchadjie et al. ^45^ improved the reactivity of granite sawdust by calcining it with Na_2_O. When this activated granite sawdust was used in a geopolymer (along with activated quartz (AQ) by 20% NaOH), the resulting geopolymer achieved a compressive strength of 29 MPa after being cured for 7 days at room temperature ^46^.

This study demonstrates the effect of inserting carbon-based additives like CFs and thermally stabilized MCC (SMCC) into FA/AQ-based geopolymer paste on the compressive strength, electrical properties, microstructure, and functionality of the produced geopolymer materials. This can transform industrial wastes into value-added materials with well-established compressive strength and electrical characteristics, indicating compatibility within FA matrix via [–Si–O…..Al–O–]_n_ networks ^47–50^.

## Materials and methods

### Materials

Fly Ash (FA) powder utilized in the production of green geopolymers was sourced as a by-product of coal combustion from Spanish electrical power station. Quartz rocks were sourced from the Eastern Desert (Egypt). Table 1 details the chemical composition of FA and quartz particles (Q). Analytical grade sodium hydroxide (NaOH, 85%) from Alpha Chemika Co. (India) served as the alkali activator for Q. Sodium silicate solution (water glass activator) with a chemical composition of 26.5% SiO_2_​, 10.6% Na_2_​O, and a modulus of 2.5 was prepared by dissolving 12.2 gm of Na_2_SiO_3_ (Merck Co. (Germany)) in 20 mL of distilled water with heating at 80 °C, achieving a density of approximately 1.4 at 25 ºC. From this prepared solution, appropriate ratios were added to the binders, as shown in Table 2. Microcrystalline cellulose (MCC) was sourced from ALFA Chemistry Co. (US). Non-sterile cotton from MISR MEHALLA A.R.E. (Egypt) served as the precursor for carbon fibers (CFs) in this study.

**Table 1:** The chemical composition of used fly ash (FA) and quartz (Q)

Oxide composition	Wt. %	
	FA	Q
SiO_2_	61.50	99.77
Al_2_O_3_	28.90	0.05
Fe_2_O_3_	3.70	< 0.01
TiO_2_	2.32	0.01
K_2_O	1.43	< 0.01
CaO	0.65	< 0.01
Na_2_O	0.23	< 0.01
MgO	0.49	< 0.01
CuO	0.07	–
ZnO	0.03	–
Cr_2_O_3_	0.03	–
SrO	0.02	–
NiO	0.02	–
MnO	0.02	< 0.01
P_2_O_5_	0.32	-
LOI	–	0.02

**Table 2:** Experimental design and batch numbers for every geopolymer with component weight ratios

		FA : AQ ratio (wt:wt)	FA : carbon additive ratio (wt:wt)	Activator: binder mass ratio	Heating temperature °C
1	FA geopolymer	1:0	1:0	0.14	70
2	FA/AQ	1:1	-	0.13	70
3	CFs(1%)@FA/AQ	1:1	1:0.01	0.27	70
4	CFs(3%)@FA/AQ	1:1	1:0.03	0.27	70
4^\^	CFs(3%)@FA	1:0	1:0.03	0.41	70
5	SMCC(1%)@FA/AQ	1:1	1:0.01	0.27	70
6	SMCC(3%)@FA/AQ	1:1	1:0.03	0.27	70

### Pretreatment for used additives

FA powder underwent calcination at 800 °C for 2.5 h to eliminate impurities and organic matter, preparing it for geopolymer formulation.

To create fine quartz particles, the quartz rock was milled for 2 h in a FRITSCH Pulverisette zirconia ball mill at a speed of 360 rpm, resulting in a fine quartz particles referred as Q. About 0.5 gm of sodium hydroxide pellets (10% wt:wt based on the weight of Q) was dissolved into 2 mL of distilled water, achieving about 6 M concentration. The quartz particles (Q) were then impregnated by droplet addition of sodium hydroxide solution and mechanically mixed by grinding. The resulting impregnated mixture was heated in a muffle furnace at 550 °C for 60 min, with a heating rate of 5 °C/min. Once cooled to room temperature, the activated-Q was stored in a sealed bag to prevent atmospheric carbonation and designated as AQ.

Microcrystalline cellulose (MCC) was thermally stabilized to improve its surface properties. This was achieved by heating MCC at 250 °C for one hour in a muffle furnace. The process yielded brown fibers, identified as stabilized microcrystalline cellulose (SMCC).

Carbon fibers (CFs) were produced following the method described by K. Abas et al. ^51^. Cotton fibers were initially cleaned with distilled water to remove any impurities, then dried in an air-oven at 105 °C for 24 h. Subsequently, the fibers underwent thermal stabilization at 250 °C for one hour in an air atmosphere. The stabilized fibers were then carbonized in a standard tube furnace, under a nitrogen flow rate of 25 mL/min. The temperature was increased from room temperature to 600 °C at a heating rate of 3 °C/min. After heating for 40 min, the resulting sample was allowed to cool to room temperature.

### Formulation procedures for green geopolymers

Geopolymers were prepared by mixing FA with or without the addition of other binders (AQ, CFs, and SMCC) in a water glass activator. FA geopolymer without additives was activated by mixing 5 gm of FA binder with 0.7 gm of water glass activator, using a mass ratio of 0.14 between the activator and binder, until paste formation. After pouring the resulting paste into a 2 cm × 2 cm × 2 cm steel mold, the mold was vibrated 25 times to remove air bubbles. The paste-filled mold was then cured at 70 °C for 48 h and subsequently allowed to rest at room temperature for 14 days.

To create the FA-based geopolymers reinforced with additives, FA (5 gm) and AQ (4.5 gm Q and ≈ 0.5 gm NaOH) binders were combined for 5 min. Then, 0.7 gm of water glass activator was introduced drop-wise, achieving a mass ratio of 0.13 between the activator (0.7 gm water glass + 0.5 gm NaOH) and binder (9.5 gm), followed by an additional 5 min of mechanical stirring till a paste formed. The assembled paste was placed into a steel mold measuring 2 cm × 2 cm × 2 cm. To eliminate air bubbles, the mold was vibrated 25 times after filling. The mold comprising the paste was initially cured at 70 °C for 48 h before being allowed to rest at room temperature for 14 days. The produced geopolymer nominated as FA/AQ. Throughout the synthesis process, the weight ratio of FA to AQ was kept at 1:1 (wt:wt).

**Fig. 1:**
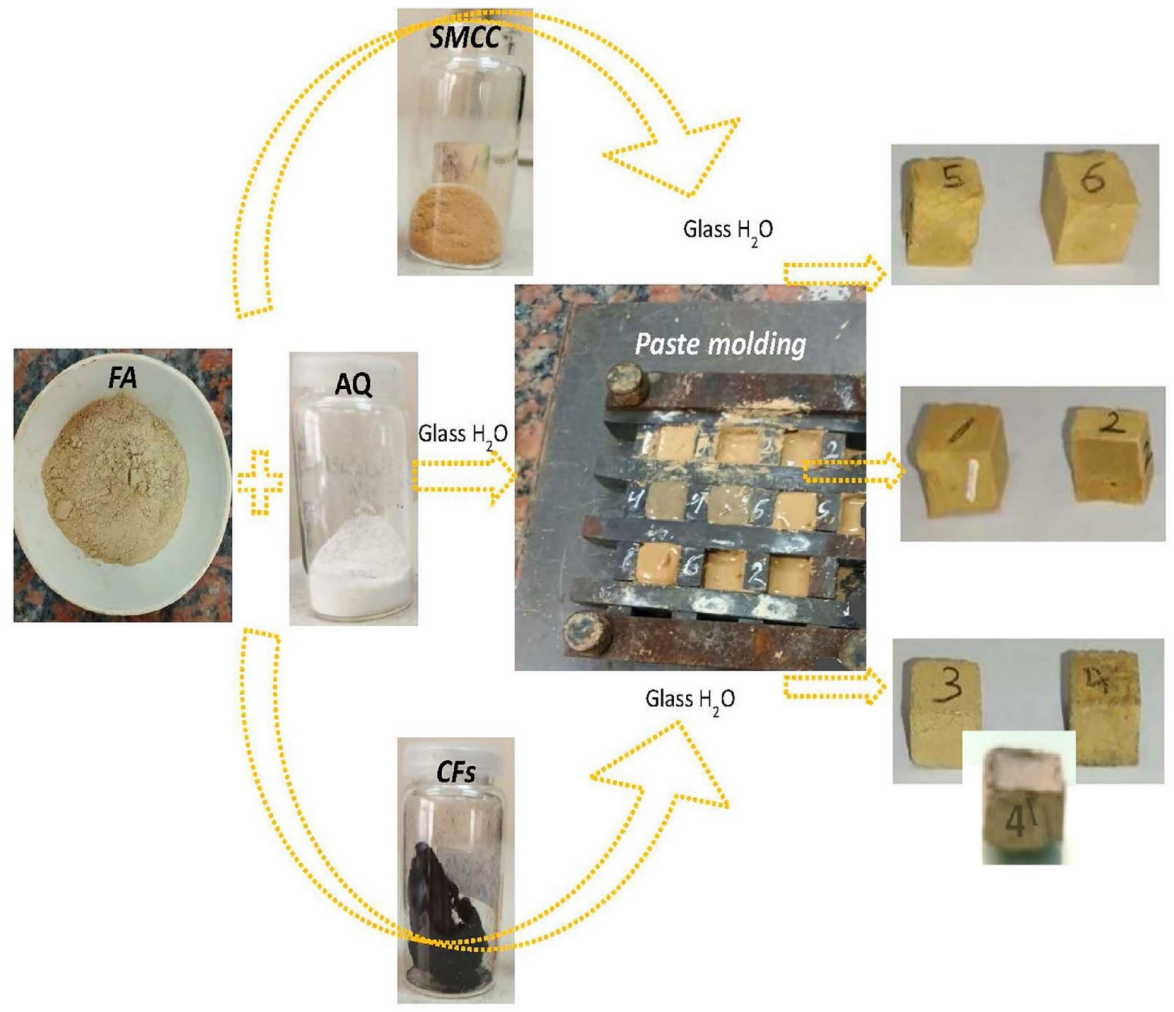
Schematic formulation of green geopolymers batched in Table 2

To prepare CFs@FA/AQ and SMCC@FA/AQ geopolymers, the previously established method was replicated, incorporating CFs and SMCC. Specifically, FA (5 gm) and AQ (4.5 gm Q and ≈ 0.5 gm NaOH) were initially mixed for 5 min. Subsequently, CFs or SMCC were separately added in two proportions (1% and 3% wt/wt) relative to FA ^52^, achieving total binder mass of 9.55 gm and 9.65 gm for the two percentages 1% and 3%, respectively. Then, 2.1 gm of water glass activator was added drop-wise until a paste formed for both concentrations, with mechanical stirring continuing for 5 min. Specimens were prepared using an activator (2.1 gm water glass + 0.5 gm NaOH) to binder (9.55 gm and 9.65 gm for every concentration) mass ratio of 0.27. The resulting paste was poured into a 2 cm × 2 cm × 2 cm steel mold. After filling, the mold was vibrated 25 times to remove air bubbles from the paste. The mold, containing the paste, underwent an initial 48-h curing phase at 70 °C, followed by a 14-day rest period at room temperature. Throughout all formulation processes, the FA to AQ weight ratio was consistently maintained at 1:1. The resulting FA-based geopolymers, labeled FA, FA/AQ, CFs(1%)@FA/AQ, CFs(3%)@FA/AQ, SMCC(1%)@FA/AQ, and SMCC(3%)@FA/AQ, were batched from 1 to 6 as obvious in Table 2 describing the experimental conditions and water glass to binder (w/b) ratio. A compatible sample from CFs(3%)@FA with zero AQ was prepared following the previously described procedure using an activator-to-binder mass ratio of 0.41 to directly depict the advantages and disadvantages of AQ on the mechanical and electrical properties of the produced geopolymer. Figure 1 presents a schematic description of green geopolymers production.

### Characterization

X-ray fluorescence (XRF) analysis, conducted on a Panalytical AXIOS WDXRF Sequential Spectrometer (2005), provided the chemical composition of FA and Q powder. Functional surface groups of the produced green geopolymers were identified via Fourier transform infra-red (FT-IR) spectroscopy employing a NICOLET 8700 spectrometer (Thermo Scientific, Loughborough, UK) and the KBr pellet technique. A Shimadzu UV–Vis spectrophotometer was functioned to record the UV–Visible absorption spectra of the prepared geopolymers at room temperature, over a wavelength range of 200–800 nm, employing distilled water as the solvent and reference. The morphology of geopolymer materials was inspected alongside a scanning electron microscope (SEM) (QUANTA FEG 250 ESEM, Japan). Chemical components of the developed samples were captured as well through energy-dispersive X-ray spectroscopy (EDX) over a voltage of 20 kV. X-ray Diffraction (XRD) analysis (PAN analytical X-Ray Diffraction equipment model X׳Pert PRO, CuKα1, λ = 0.1542 nm) was performed on the obtained geopolymer materials at room temperature, applying monochromator settings of 45 kV, 35 mA, scanning speed 0.04°/sec, and 2θ = 0–60 °C. Compressive strength was evaluated following the ASTM C 1424–19 standard using a Tinius Olsen Universal testing machine (model 25ST, UK) at a crosshead speed of 0.5 mm/min. The geopolymers were ground into powder and pressed into 1-inch diameter, 1-inch thick pellets using a stainless-steel mold (10 kN force), then analyzed for electrical conductivity using a YHP4192A impedance analyzer (Yokokawa, Hewlett Packard Japan Ltd., Tokyo, Japan) at frequencies from 0.0 Hz to 3.0 MHz.

## Results and discussion

### Structural analyses

#### FT-IR analysis

**Fig. 2:**
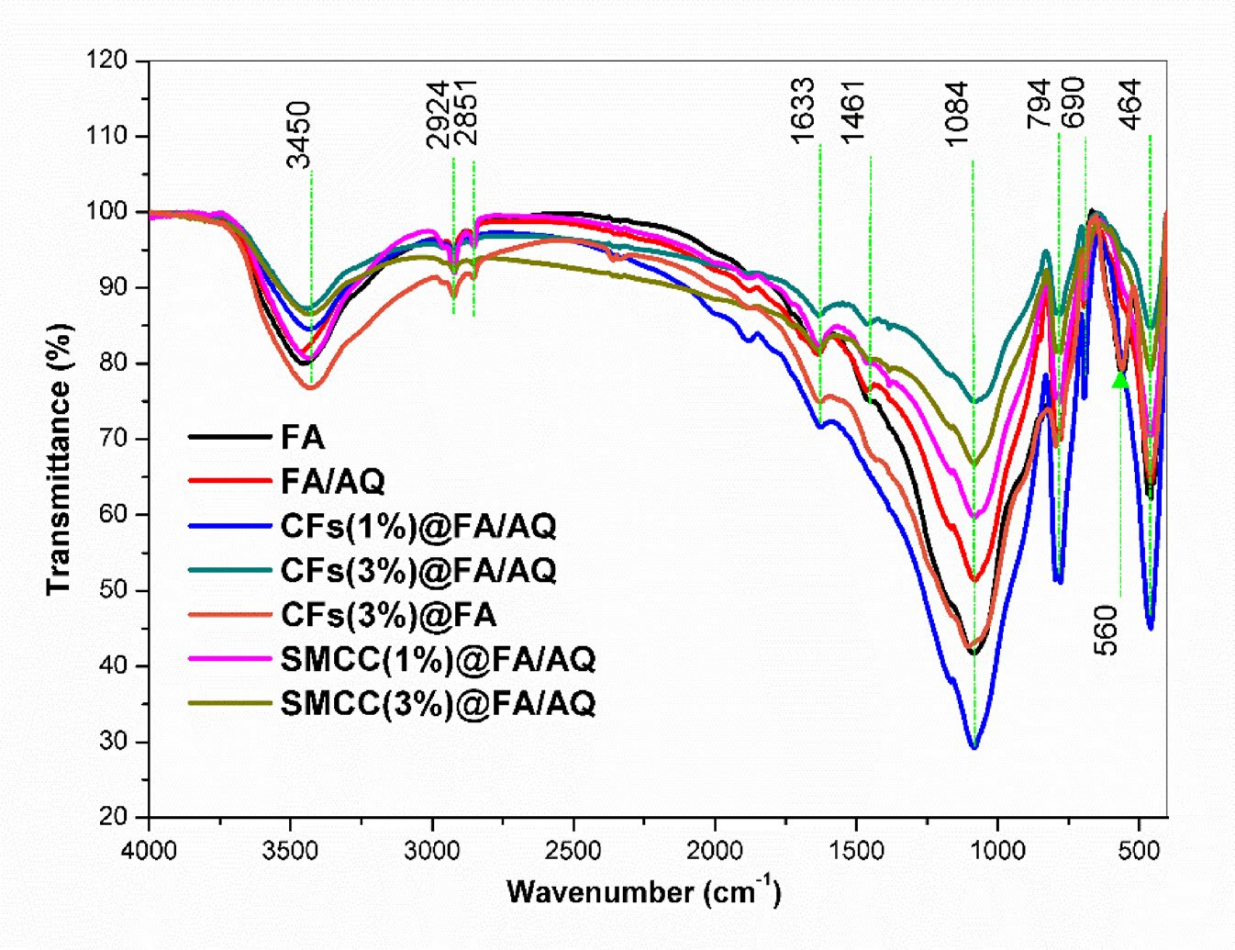
FTIR spectra of FA-based geopolymers

Figure 2 reveals the chemical functional groups typical of FA across all synthesized geopolymer materials. The broad peak detected between 3700 and 3300 cm^-1^ is attributed to the stretching and bending vibrations of surface hydroxyl (-OH) groups linked to silanol (-Si–OH) functionalities. These vibrations become prominent after calcination pretreatment ^53^. The bending vibration of the -OH groups from adsorbed water molecules is credited to the band at about 1633 cm^−1^. The symmetric and asymmetric stretching vibrations of the silicate Si–O-T (T = Si or Al) are responsible for substantial bands at 794 and 1084 cm^−1^, respectively ^53^. Rather, a low-intensity peak emerges at a higher wavenumber, 1461 cm^-1^, suggesting that the FA structure after calcination contains fewer silica and alumina minerals ^54–56^. Al–O–Al/Si bending vibrations (mullite) are accountable for the bands around 464 and 560 cm^−1^ ^57^. Additives in the geopolymer matrix are frequently identified by their own distinct bands or by how they affect pre-existing peaks ^58^. The FTIR patterns of the created FA-based geopolymers showed that, following blending with AQ, CFs, and SMCC, the primary peaks in the FA structure shifted to lower band intensities. The typical band for Al–O–Al/Si bending vibrations (mullite) around 560 cm^−1^ is absent. Instead, the symmetrical bending vibration of Si–O, distinctive to AQ, is recognized at 690 cm^−1^. Upon AQ introduction, the former band at the FA exhibits only slight intensity because the amorphous portion of FA transformed into the crystalline phase ^59^. The band at approximately 1461 cm^-1^ in the geopolymer specimens is therefore ascribed to asymmetric carbonate stretching, suggesting mild carbonation of the AQ, possibly due to air exposure ^60^.

The degree of polysialylation, the primary fingerprint for the geopolymer matrix, is defined by distinct aluminosilicate bands at 794 cm^-1^ ^61^ and 1084 cm^-1^ ^59^. When CFs and SMCC are integrated into a geopolymer paste, these bands appear with lower intensity due to the dissolution of aluminosilicate during the geopolymer reaction. Furthermore, other characteristic FA/AQ bands may shift to lower intensity. This can be accomplished because the C–H stretching of methyl and methylene groups appears in the range of 2851–2924 cm^-1^, and the aromatic skeletal vibration of C = C, assigned to CFs, is inserted at 1633 cm^-1^ for CFs@FA/AQ geopolymers ^62^. However, the characteristic band peaks of CFs(3%)@FA geopolymer are similar to neat FA geopolymer owing to the removal of the crystalline phase of AQ. Additionally, the depiction of C–O–C pyranose ring vibration causes a decrease in the characteristic band intensity at 1084 cm^-1^ for SMCC@FA/AQ geopolymers ^63^.

#### UV/visible analysis

**Fig. 3:**
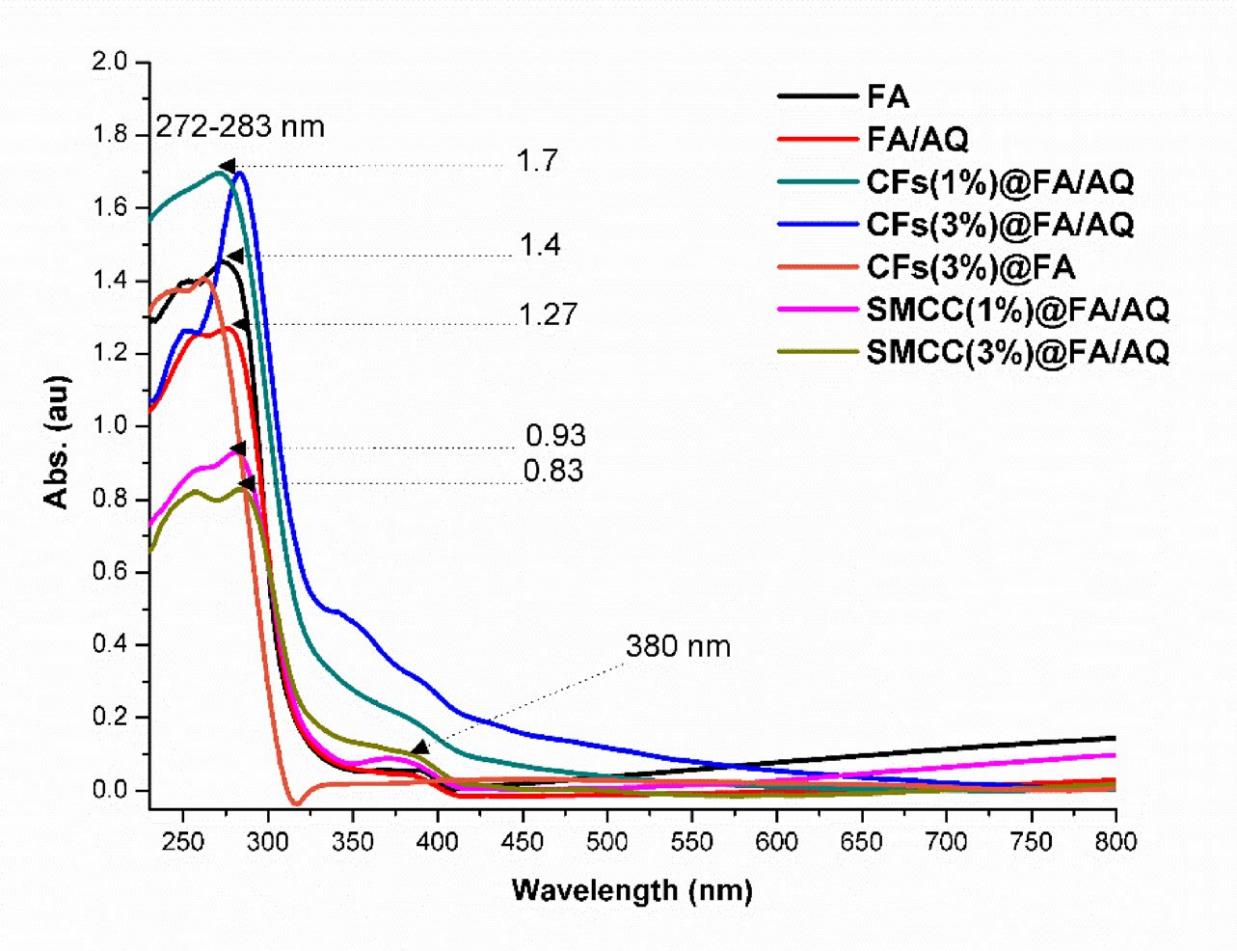
UV/Vis spectra of prepared FA-based geopolymers

UV/Vis measurements at a particular wavelength can characterize produced geopolymers by inspecting light absorption in the ultraviolet region of 200–800 nm. This method provides insights into their optical characteristics, which are influenced by their structural and chemical constituents. These studies enable researchers to better comprehend the interaction of FA with other constituents in the geopolymer matrix, including the influence of additives on the composite’s properties. Figure 3 demonstrates that FA-based geopolymers exhibit no significant absorption in the visible region (400–800 nm), with dominant absorption peaks observed exclusively within the UV range 200–400 nm ^64^.

**Fig. 4:**
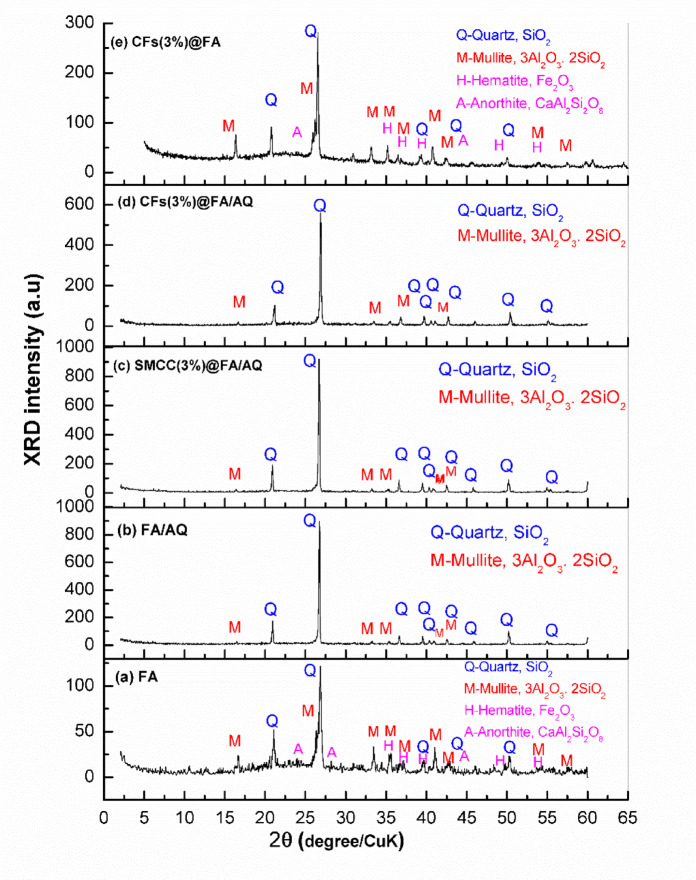
XRD patterns of FA-based geopolymers

Interpretation of the acquired UV/Vis spectra reveals that FA, FA/AQ, SMCC(1%)@FA/AQ, and SMCC(3%)@FA/AQ geopolymer materials exhibit a substantial absorption band at a maximum absorption wavelength of 272 nm and a modest intensity band at 380 nm. Compared to the neat FA geopolymer absorbance value of 1.4, the maximum distinctive band absorbance for FA/AQ, SMCC(1%)@FA/AQ, and SMCC(3%)@FA/AQ record values 1.27, 0.93, and 0.83, respectively. The low absorbance value may be attributed to the structural features of AQ, demonstrating a high degree of structural order with a minimal amorphous phase, as apparent in XRD evaluation (Fig. 4b). As evidenced by the detection of an amorphous matrix in the XRD sample, the structural disorder of the synthesized neat FA geopolymer is also accountable for the elevated absorbance value ^65^. Conversely, the CFs(1%)@FA/AQ and CFs(3%)@FA/AQ specimens exhibit the highest absorbance value of approximately 1.7 at the maximum characteristic peak. This indicates the presence of an amorphous geopolymeric matrix accompanied by residual semi-crystalline phases. The disappearance of the absorption band at 380 nm, coupled with the observed shift of the principal absorption peak from 272 to 283 nm and 262 nm for the CFs(3%)@FA/AQ and CFs(3%)@FA, respectively, is consistent with FTIR spectral data, thereby substantiating the successful chemical interaction and integration of CFs within the FA-geopolymeric network ^66^.

Following comprehensive FTIR and UV/Vis spectroscopic analyses conducted on the seven synthesized geopolymer systems, five green geopolymer formulations of (FA, FA/AQ, CFs(3%)@FA/AQ, CFs(3%)@FA, and SMCC(3%)@FA/AQ) were identified as candidates for advanced investigation due to their promising physicochemical characteristics relevant to mechanical and electrical applications.

#### XRD analysis

A qualitative phase study was carried out with the aid of an X-ray diffraction (XRD) and revealed in Fig. 4. The XRD data in Fig. 4a highlight distinct crystalline phases alongside the amorphous glass mineral components that constitute the fly ash. The neat FA geopolymer sample features an asymmetric amorphous hump with a wide reflection at 2θ = 20–25°, analogous to the amorphous aluminosilicate glass phase ^67^. In the geopolymerization process, amorphous aluminosilicate is the principal source of reactive precursors, impacting both the material’s ultimate strength as well as the reaction kinetics ^68^. Remarkably, this amorphous hump was more attenuated in the subsequent FA-based geopolymers due to the dissolution of aluminosilicate during the geopolymer reaction and the formation of a more crystalline quartz phase. Using Powder Diffraction Files (PDFs) from the Inorganic Crystal Structure Database (ISCD), the fly ash’s X-ray diffraction patterns, as shown in Fig. 4a, disclose four main crystalline phases. These phases are quartz (SiO_2_) (Q-PDF no. 01- 083–2468) ^69^, mullite (3Al_2_O_3_.SiO_2_) (M-PDF no. 01–074-2419) ^70^, hematite (Fe_2_O_3_) (H-PDF no. 01–086-0550) ^71^, and anorthite (CaAl_2_Si_2_O_8_) (A-PDF no.00–041-1486) ^72^. The quartz and mullite minerals indicate the presence of silica and alumina. Quartz in fly ash is normally inert, whereas non-reactive mullite progressively contributes to the geopolymerization process. Hematite, a type of iron oxide, is present in low content, remains inactive even after the geopolymerization reaction, and has no effect on the material’s ultimate characteristics ^73^.

By comparing the XRD spectra of the neat FA geopolymer with those of other green geopolymers, considerable information may be gleaned from the XRD patterns (Fig. 4b, c, d, and e). Firstly, the addition of AQ to FA geopolymer creates more crystalline and defined quartz peaks with high intensity. These peaks support the quartz’s trigonal structure with space group P3_2_21 and demonstrate the destruction of alkali-silicate glasses, confirming their contribution to the geopolymerization reaction with partial transition into the crystalline structure of quartz ^74^. Stutzman et al. ^75^ linked high quartz peak intensities to preferred orientation effects with increasing quartz content rather than actual structural alterations. Secondly, tiny mullite peaks with low intensities are still detectable in the X-ray diffraction patterns of geopolymers, pointing that the majority of the fly ash was involved in the geopolymerization event. However, a small portion remained unreacted or unincorporated during the reaction. The addition of SMCC to FA/AQ geopolymer, SMCC(3%)@FA/AQ (Fig. 4c), results in the intensity and position of the diffraction peaks coinciding with those of the FA/AQ geopolymer (Fig. 4b). This verifies the presence of the identical mineral phases, suggesting a distinct material response, as found by Guzm´an-Aponte et al. with the addition of TiO_2_^76^.

For the CFs(3%)@FA/AQ and CFs(3%)@FA geopolymer samples (Figs. 4d and e), an amorphous background was detected at the peaks’ baseline, likely due to the introduction of CFs into the geopolymer. The quartz peak intensities were distinguishable, decreasing as a sign of compositional change and a reduction in the crystalline quartz phase. This suggests a higher polymerization process compared to other FA-based geopolymers and absence of AQ in CFs(3%)@FA sample.

The Scherrer equation (Eq. 1) was employed to evaluate the particle size of the geopolymer composites used in this investigation.

1 $$Lc = \frac{K.\lambda }{{\beta .\cos \theta }}$$

where K is the crystallite form factor (assuming 0.9), λ is the source’s X-ray wavelength, which is 1.542Å for Cu radiation, θ is the diffraction angle, and β is the full-width at half-maximum (FWHM). The coherence lengths (Lc) determined for FA, FA/AQ, CFs(3%)@FA/AQ, CFs(3%)@FA, and SMCC(3%)@FA/AQ geopolymers are 38.6 nm, 57.5 nm, 41.9 nm, 33 nm and 49.2 nm, respectively. The wide, amorphous peaks of aluminosilicates are the reason behind the small crystalline size of FA and CFs(3%)@FA geopolymers, whereas the sharp peaks of the crystalline quartz phase suggest a larger crystalline size for other prepared FA-based geopolymers.

### Microstructural analyses

**Fig. 5:**
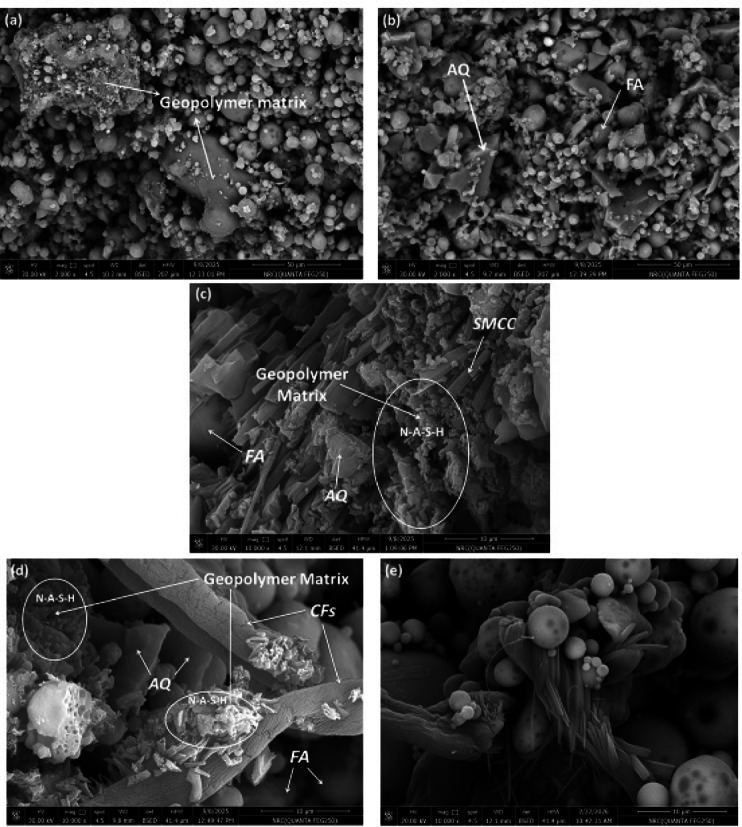
SEM micrographs for; (**a**) FA, (**b**) FA/AQ, (**c**) SMCC(3%)@FA/AQ, (**d**) CFs(3%)@FA/AQ, and (**e**) CFs(3%)@FA geopolymers (*representing the network of N-A-S–H gel*)

**Fig. 6:**
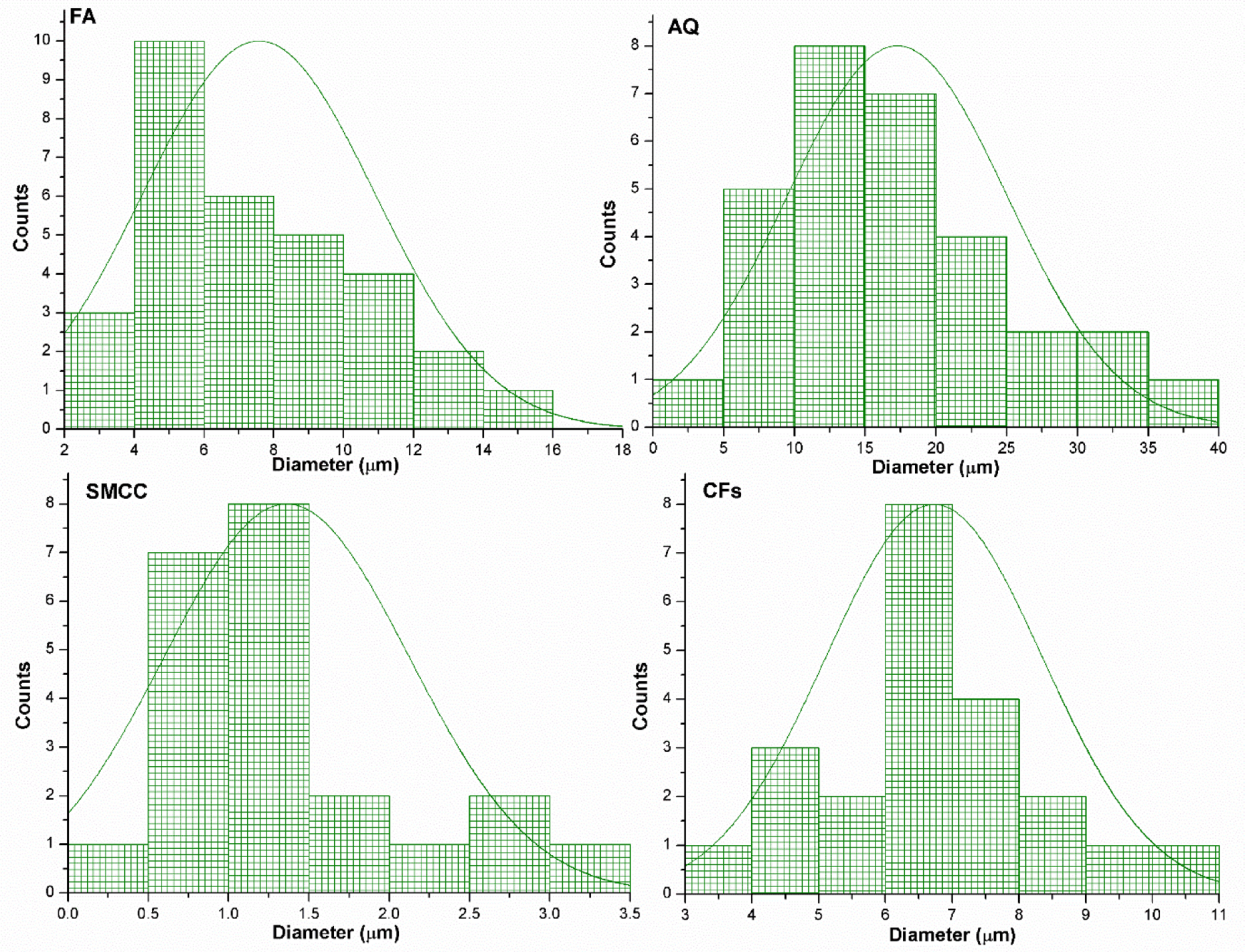
Diameter distribution curves for used additives for geopolymer preparation

**Fig. 7:**
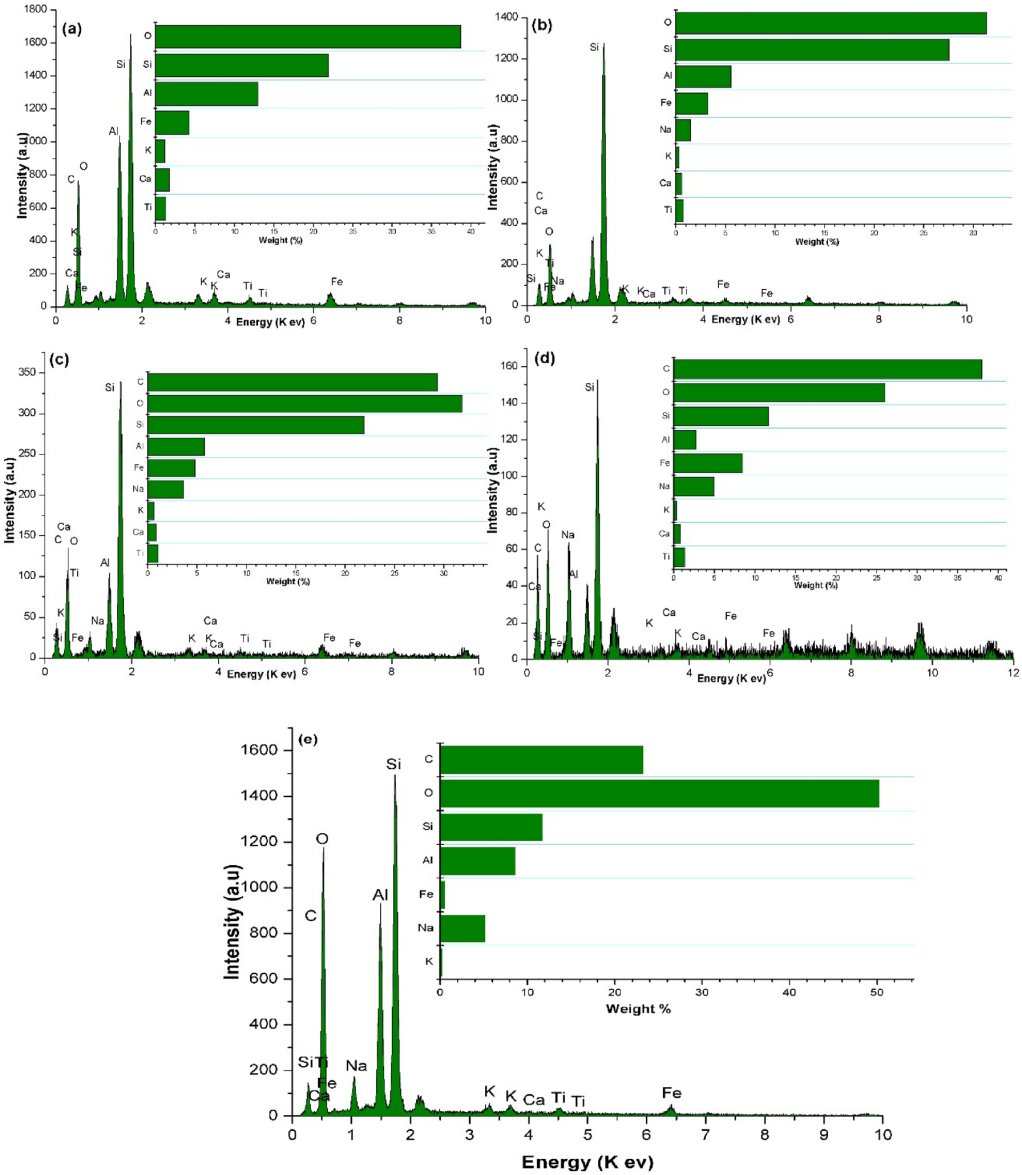
EDX analysis of; (**a**) FA, (**b**) FA/AQ, (**c**) SMCC(3%)@FA/AQ, (**d**) CFs(3%)@FA/AQ, and (**e**) CFs(3%)@FA geopolymers

The developed samples were examined using SEM, diameter distribution curves for used additives, and EDX techniques to investigate their morphological features as obvious from Figs. 5, 6 and 7, respectively. The SEM study indicates significant alterations underwent in the fly ash geopolymer’s 3D microstructure as a result of the formation of a geopolymer matrix based on various utilized additives. Microscopic analysis often reveals that geopolymer matrices are composed of spherical particles and glassy, flake-like structures, forming a polymerized aluminosilicate network which apparent in all prepared geopolymers. In spite of absence of cracks, presence of voids and cavities in the produced geopolymers affect directly its mechanical and electrical characteristics. Figure 5a depicts the silky texture of small to medium-sized vitreous spherical, rounded particles of unreacted FA in the FA geopolymer with an average diameter of 7.6 ± 3.3 µm; this circular morphology may boost the composition’s functionality. Additionally, the morphology displays clusters of fragmented, dense particles, which could result from the coal combustion’s quick cooling phase. The presence of unreacted discrete FA particles implies the inadequate dissolution of the aluminosilicate and suggests low strength in the FA geopolymer. With the inclusion of AQ (Fig. 5b), the main morphological disparity between FA and FA/AQ geopolymers is the difference in the physical form of their unreacted particles, representing incomplete geopolymerization process. The microstructure becomes more complex with the development of a gel matrix. The FA particles are smaller, while AQ grains are large, neat, and angular–shaped particles with a mean diameter of 17.3 ± 7.8 µm. The jagged shape of AQ and its nearly invariable highly crystalline nature, which influences the durability and mechanical strength ^77^, are attributed to the crushing, milling, and activation procedures utilized during manufacture. Thus, based on earlier researches ^78,79^, quartz grains enhance the density and decrease the porosity of the geopolymer composite in addition to its role in the geopolymerization activity.

For carbon and cellulose/geopolymer materials, SMCC(3%)@FA/AQ, CFs(3%)@FA/AQ, and CFs(3%)@FA, the commencement of clear geopolymer matrix formation is evident. Hydroxyl group (OH^−^) attack initiated a layer-by-layer dissolution of the FA particles, breaking them down into small aluminum and silicon species ^80^. Subsequently, the dissolved species underwent aggregation, forming particles with a three-dimensional network of N-A-S–H linkages (Figs. 5c, d and e). This network is responsible for the improved mechanical strength of the material. The uniform N-A-S–H bonding within the geopolymeric gel led to the formation of densely packed, flake-shaped particles. Consistent with previous findings ^81^, the reaction of aluminosilicates with a Na-based alkaline solution resulted in the formation of N-A-S–H gel. This geopolymerization process involves the dissolution of aluminosilicates, producing SiO_4_ and AlO_4_ tetrahedra that subsequently link via oxygen bonds to create Si–O-T (T = Si or Al) structures.

In SMCC(3%)@FA/AQ geopolymer (Fig. 5c), bundles of erratic, micro-sized, rod-shaped SMCC with a mean diameter of 1.36 ± 0.76 µm are observed with fewer particles from unreacted FA and AQ. In contrast to the lack of matrix-fiber coherence, excellent adhesion is observed between the cellulosic rods and the matrix, which undoubtedly affects the mechanical characteristics of the geopolymers. The incorporation of CFs into the geopolymer matrix, CFs(3%)@FA/AQ (Fig. 5d), reveals some smooth unreacted fibers with uniform dimensions and a mean diameter of 6.7 ± 1.6 µm, besides lower number of unreacted FA particles and AQ grains due to their breakdown. It is worth noting that the including of CFs in the geopolymer accelerates the reaction rate of geopolymer matrix production, assists in maintaining structural integrity, and thus has a beneficial impact on boosting the composite’s bending strength. By comparing CFs(3%)@FA/AQ (Fig. 5d) with CFs(3%)@FA (Fig. 5e) geopolymers in absence of AQ, more voids and cavities are presented which can affect directly on electrical and mechanical properties without any change in unreacted species diameter distribution.

The EDX results (Fig. 7) demonstrate variations in the compositional elements of the prepared green geopolymers. The EDX spectra of the composites depict prominent peaks belong to O, Si and Al, confirming Si and Al as the primary constituents of the FA, consistent with the composition of quartz and mullite phases. Additional peaks for Fe, K, Ca, Na, and Ti were also found; these elements are most likely present as trace amounts within the FA. The greatest Si value is seen in the FA/AQ geopolymer, which is anticipated as the inclusion of AQ increases the percentage of the quartz crystalline phase. These findings are consistent with those obtained using XRD analysis. A minor quantity of carbon in the FA and FA/AQ geopolymers might be a contaminant during sample preparation for SEM examination. The carbon percentage increased significantly in CFs(3%)@FA, CFs(3%)@FA/AQ, and SMCC(3%)@FA/AQ geopolymers due to the addition of carbon sources of CFs and SMCC. Detecting areas with lower oxygen weight (%) for SMCC(3%)@FA/AQ geopolymer could indicate a denser, more amorphous N-A-S–H gel, leading to enhanced compressive strength. However a higher oxygen weight (%) of CFs(3%)@FA could be attributed directly to the loose geopolymer structure with the presence of cavities, which can lead to high electrical conductivity and lower mechanical strength.

### Compressive strength of prepared geopolymer matrices

**Fig. 8:**
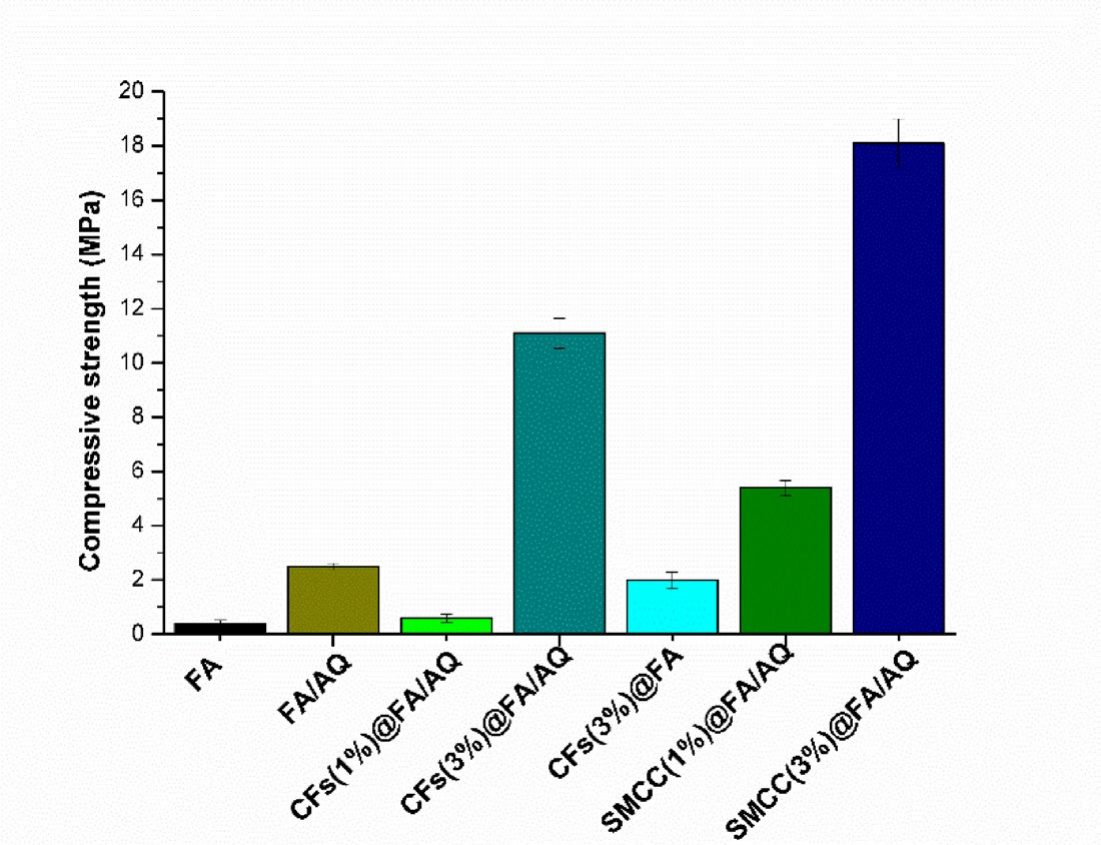
Compressive strength of FA-based geopolymers with various additives

This section of the research utilized neat FA geopolymer reinforced with various additives (FA/AQ, CFs(1%)@FA/AQ, CFs(3%)@FA/AQ, CFs(3%)@FA, SMCC(1%)@FA/AQ, and SMCC(3%)@FA/AQ), demonstrating the compressive strength values of geopolymer matrices as presented through Fig. 8. The neat FA geopolymer exhibits the lowest strength, approximately 0.4 MPa, compared to other matrices. This is attributed to inadequate alkaline activation, leading to incomplete geopolymerization, or poorly managed curing conditions, resulting in slow reaction rates. Additional factors comprise impurities such as CaO content in the fly ash, which reduce strength ^82,83^. For FA/AQ, incorporating AQ improves the mechanical properties by functioning as a fine filler, compacting, and densifying the matrix ^84,85^. The strength rises to 2.5 MPa as illustrated in Fig. 8. This is a result of the creation of Si-centered radicals and non-bridging oxygen vacancies on the quartz surface, which promote geopolymerization ^84^. Furthermore, the highly active Si particle can substitute the Al particle in the geopolymer matrix, resulting in the formation of a Si-rich gel around quartz. Impact of AQ on the mechanical strength of produced geopolymer evident in the CFs(3%)@FA geopolymer as its absence reduce its compressive strength to 1.62 MPa. While for CFs@FA/AQ, the strength value improves with the integration of CFs compared to the plain sample of neat FA geopolymer. The fibers enhance the material’s ability to absorb energy and resist blasts, simultaneously serving as nucleation sites that encourage the geopolymerization process to preserve elevated compressive strength. Raising the CFs percentage from 1 to 3% in CFs(1%)@FA/AQ and CFs(3%)@FA/AQ geopolymers increases strength values from 0.6 to 11.1 MPa. This is potentially attributed to the insufficient dispersion of the small quantity of CFs at 1% ^86^, while the higher percentage exhibits strong compatibility with the geopolymer matrix with the formation of dense, amorphous N-A-S–H linkage ^87^. In geopolymer matrices infused with SMCC, SMCC(1%)@FA/AQ exhibits a strength of 5.4 MPa, while the matrix doped with a higher SMCC content (SMCC(3%)@FA/AQ) reaches 18.1 MPa. This is due to the following factors: a) the incorporation of SMCC creates moisture migration pathways and acts as internal curing agents, which can enhance the internal structure of geopolymers, facilitating geopolymerization reactions ^88^; b) heightened hydrogen bonding reactions may occur between SMCC and the geopolymerization matrix owing to SMCC’s high surface reactivity ^89^; c) SMCC aids in filling micro-voids, decreasing porosity, and encouraging dense, amorphous N-A-S–H network coordination, thereby enhancing mechanical properties ^90^. All of these possibly raise the non-evaporable water content ^91^, where a portion of the water chemically reacts and becomes “bound” to these new compounds. There is a strong correlation between non-evaporable water content and concrete strength development, as non-evaporable water is a byproduct of the hydration process that binds the paste together and increases its strength ^92^. Higher non-evaporable water content generally indicates a higher degree of hydration, which leads to a more compact and stronger hardened paste with lower porosity, increasing compressive strength over time. Thus, various additives can influence the mechanical strength of geopolymer materials ^93–96^, as depicted from Table 3.

**Table 3:** Comparison of the mechanical strength of our optimum fabricated geopolymers with various recorded samples

Geopolymer	Compressive strength (MPa)	References
FA	0.4	This study
FA geopolymer paste	0.96–3.64	^97^
Low-calcium fly ash-based Geopolymer mortar	1.6	^82^
CFs(3%)@FA	1.62	This study
FA/AQ	2.5	This study
Geopolymer foam concrete (GFC)	1–10	^98^
Glass foam	6.4–6.8	^99^
Białystok-based geopolymer	7.2	^100^
Aerated Geopolymer block GPB2 M1	10.95	^101^
CFs(3%)@FA/AQ	11.1	This study
Cotton fibers (2.1%)/FA	13	^39^
MK geopolymer	15	^7^
MWCNTs (2%)/alkali-activated slag	15	^102^
Metakaolin geopolymer/CNTs	17	^86^
SMCC(3%)@FA/AQ	18.1	This study

### Electrical properties of generated geopolymers

#### Electric-conductivity measurement

**Fig. 9:**
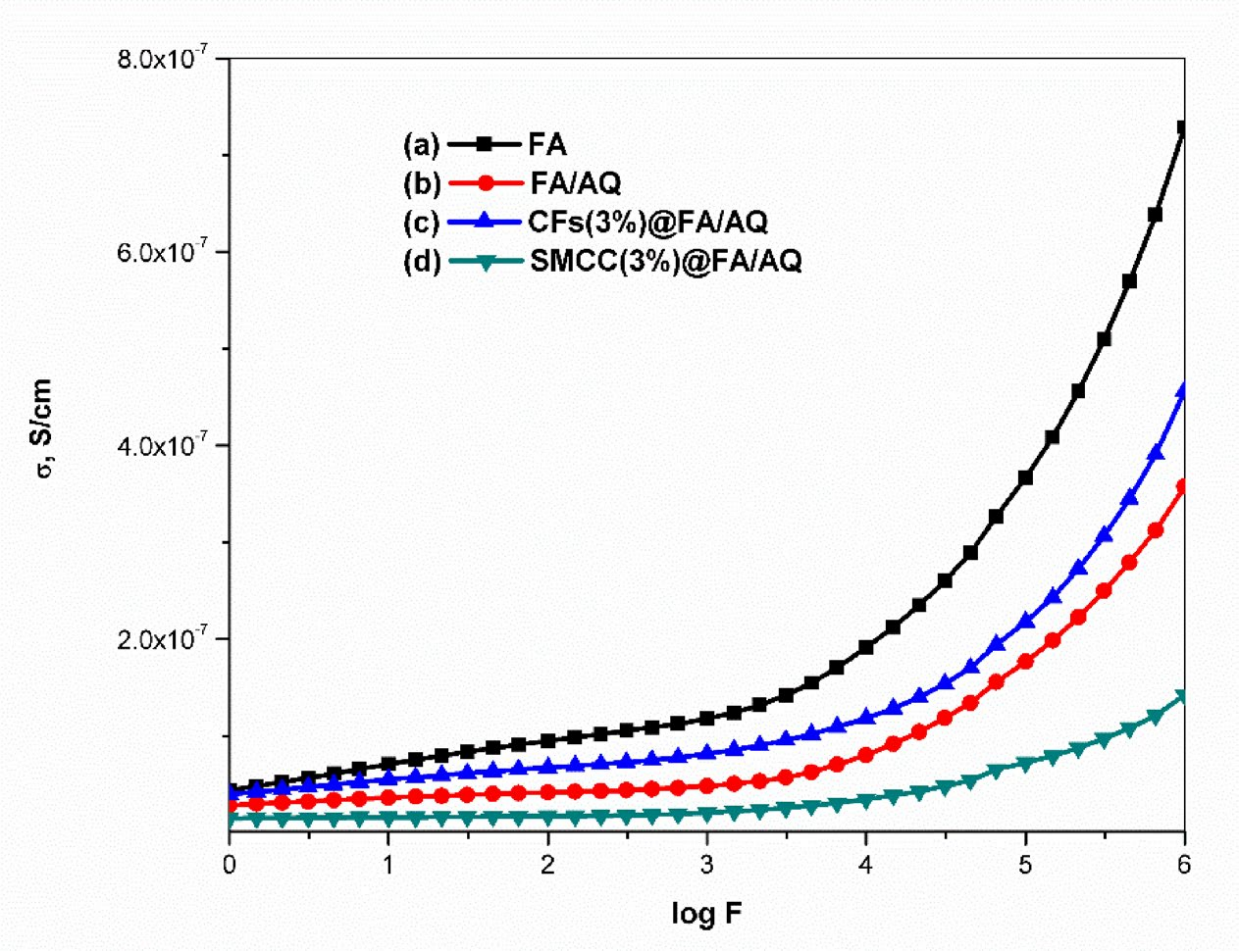
Electrical conductivity as a function of frequency for the synthesized geopolymers

Figure 9 illustrates the changes in electrical conductivity (σ, S/cm), across the frequency (F, Hz) range of 0.0 Hz to 3.0 MHz. These measurements, recorded at room temperature for the selected samples, were obtained for FA-based geopolymers incorporating AQ, CFs, and SMCC. Conductivity alteration occurs due to the reorganization and rearrangement of charges at the sample’s interface in response to an external electric field ^103^. The conductivity measurements show a non-linear frequency response due to electron hopping and interfacial polarization ^104,105^. The σ values increased steeply with frequency in all samples, particularly at lower frequencies (up to 10^3^ Hz). This increase became even more pronounced in the higher frequency range of 10^3^–10^6^ Hz. Due to the reduction in polarization with frequency, the electrical conductivity of the samples rises with frequency. Conductivity and polarization are often inversely related, as polarization is a phenomenon that can hinder or diminish a material’s conductivity. Polarization happens when charge separation accumulates at a boundary, counteracting the applied electric field and diminishing the overall capacity of charges to move within the material ^104,105^. It contends that the rise in conductivity with higher frequency results from a continuous reduction in resistance ^106^. Figure 9a presents the σ of the FA geopolymer sample as a function of frequency, reaching a maximum value of 7.3 × 10^–7^ S/cm at frequency 10^6^ Hz. This aligns with the findings from the XRD and microstructure, which indicate that the matrix exhibiting the least crystallinity produces high conductivity ^107^. These results match well with the research conducted by Sakonwan et al. ^107^.

Figure 9b depicts the conductivity of FA/AQ geopolymer. A reduction in σ value (3.6 × 10^–7^ S/cm) was observed compared to the FA geopolymer, which can be attributed to densification effect of AQ. Silica is classified as a wide band gap semiconductor because it lacks free electrons, resulting in extremely low conductivity under typical conditions. Nonetheless, its influence on conductivity varies and is contingent upon the particular type of silica and the surrounding environment. An abundance of silica may impede electron movement, resulting in reduced electrical conductivity ^108^. However filling FA/AQ geopolymer with CFs, CFs(3%)@FA/AQ (Fig. 9c), increased the conductivity by a factor of 1.3 reaching a value of 4.6 × 10^–7^ S/cm. This increase resulted from heightened electrostatic repulsion and weak Van der Waals attractive forces among the CFs, causing uniform dispersion of the fibers within the matrix and improved electrical conductivity. Thus, the conductivity of the composites increased proportionally with CFs addition ^109^. Some literature exhibits lower conductivity values with CNTs addition compared to the FA sample, which may result from inadequate dispersion of the tubes. This may be attributed to the tendency of nanomaterials to stay clustered due to the very strong Van der Waals attractive forces, which can influence the composite performance ^110^.

Incorporating cellulose in geopolymer composites, SMCC(3%)@FA/AQ (Fig. 9d), exhibits a lower electrical conductivity (σ = 1.4 × 10^–7^ S/cm) compared to other matrices. This reduction is likely due to the thermal removal of polar hydroxyl groups from cellulose, a phenomena confirmed by EDX measurement. The inherent characteristics of cellulose, including its crystalline structure, dimensions, and nature of its side groups, are crucial elements that influence its electric properties ^111,112^. Additionally, electrical conductivity is potentially reduced by the presence of dense, amorphous N-A-S–H links, which create a tortuous path that hinders electric current flow and lowers ion diffusion rates. The addition of silica amplifies this effect, further limiting ion mobility and potentially reducing electrical conductivity.

#### Dielectric properties measurement

**Fig. 10:**
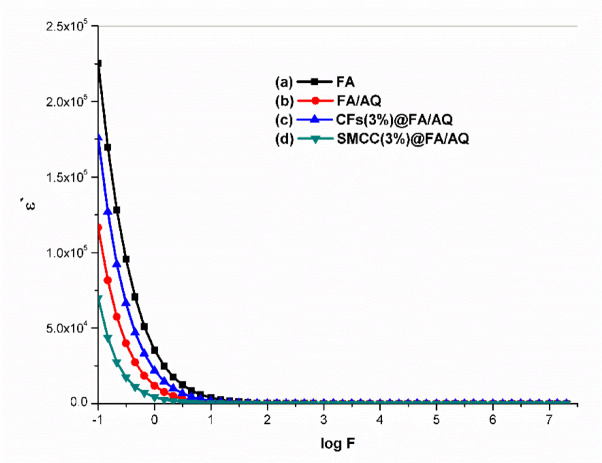
Dielectric constant variation as a function of frequency for the synthesized geopolymers

**Fig. 11:**
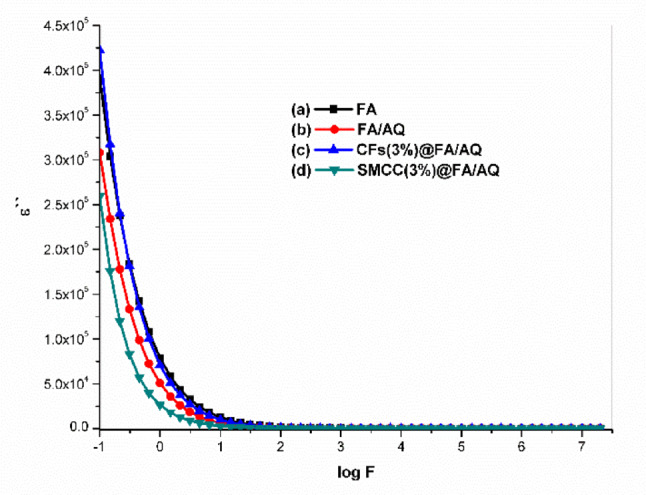
Dielectric loss variation as a function of frequency for the synthesized geopolymers

The dielectric relaxation mechanism is affected by molecular groups bound to the polymer structure ^113^, as well as factors like frequency, moisture levels, and geopolymer composition ^112^. These factors ultimately influence the geopolymer’s dielectric characteristics. Figures 10 and 11 illustrate the dielectric constant (ε΄) and dielectric loss (ε΄΄), respectively, as a function of frequency at room temperature for the prepared geopolymers. Both ε’ and ε’’ exhibit elevated values in the low-frequency region, which gradually decrease as the frequency increases. In the high-frequency regime, the influence of the applied external field on these parameters becomes independent. The elevated dielectric response observed in the low-frequency regime is generally attributed to interfacial polarization effects, which may arise within the composite material itself or at the interface between the sample and the electrodes ^114^. Furthermore, the relatively slow variation of the applied alternating current (AC) field at these frequencies allows molecules sufficient time to rotate and align their dipoles with the field’s direction ^115^. However, high frequencies hinder molecular reorientation, causing a decrease in ε′ and ε′′ due to polarization relaxation ^116^. The dielectric constants were also affected by the addition of AQ. The ε′ values decreased twofold with AQ inclusion in the FA/AQ geopolymer (Fig. 10b) compared to the FA geopolymer (Fig. 10a), due to the non-polar nature of SiO_2_. The ε′ value decreased for SMCC(3%)@FA/AQ geopolymer (Fig. 10d). This decrease could be attributed to an electrically conductive phase present in SMCC. Moreover, the dielectric property of cellulose fibers is strongly affected by humidity; higher humidity leads to lower ε′ ^112^. The conductive nature of carbon in CFs(3%)@FA/AQ reduces the sample’s insulation and the dielectric constant properties (Fig. 10c) compared to FA geopolymer, while dielectric loss increases, representing more energy dissipation (Fig. 11c).

**Fig. 12:**
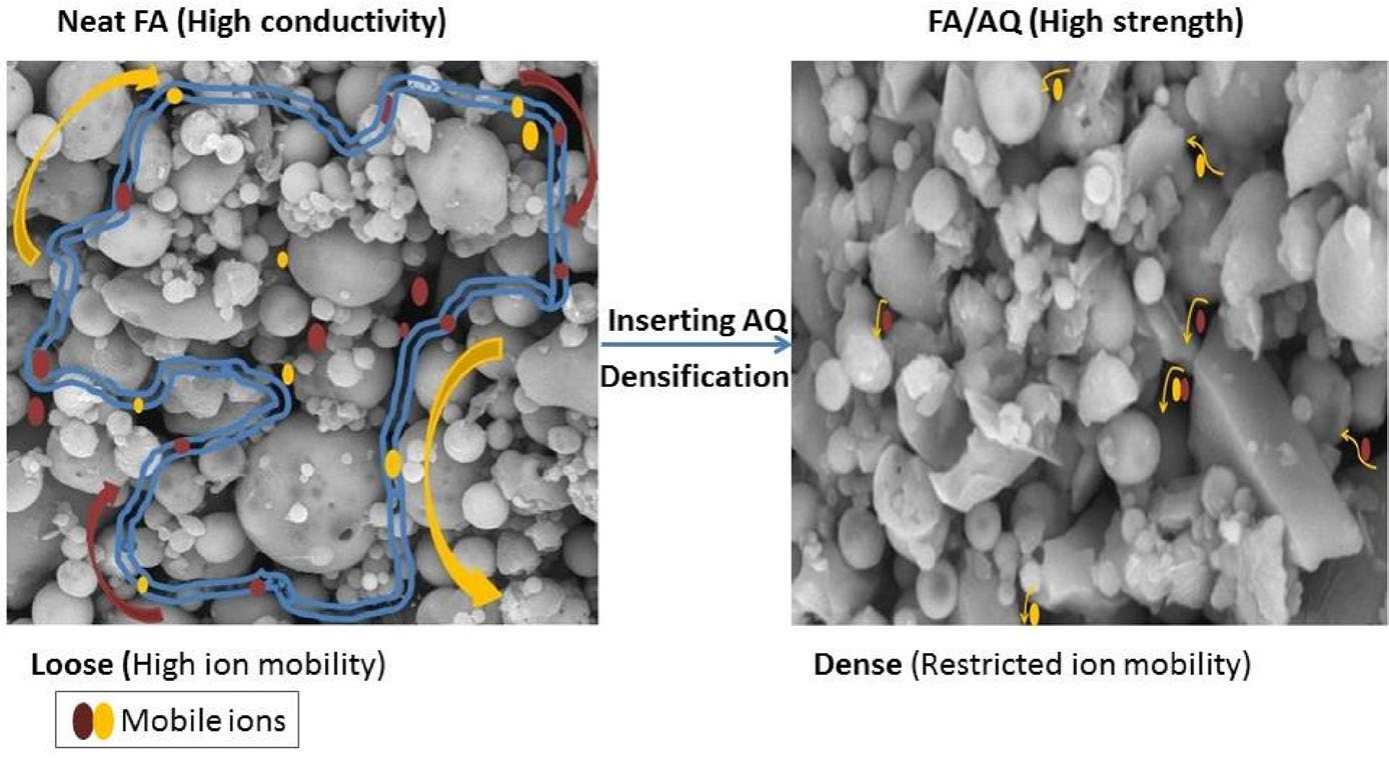
Effect of AQ additive on ion mobility and network structure

Figure 12 establishes a comprehensive structure–property model of AQ, systematically discussing its function in pore structure, ion mobility, and matrix densification, influencing both mechanical and electrical performance. In the neat FA geopolymer, incomplete polymerization, indicated by lower mechanical strength, produces a more porous and interconnected structure. This network maintains a higher volume of voids saturated with mobile Na^+^/OH^-^ ions derived from the unreacted alkaline activator. Figure 12 demonstrates that the porous structure of neat FA geopolymer promotes a percolation path for current by allowing Na^+^/OH^-^ ions from the unreacted activator to move freely. The abundance of mobile ions creates a high-conductivity environment despite the material’s reduced strength. However, integrating AQ into FA-based geopolymer leads to significant matrix densification and reduced porosity, resulting in high mechanical strength. This densification restricts ion mobility, leading to high strength and low conductivity despite the presence of the same initial activator concentration ^117,118^. Thus, the lower conductivity of the FA/AQ sample reflects improved structural densification rather than merely the insulating character of AQ.

**Fig. 13:**
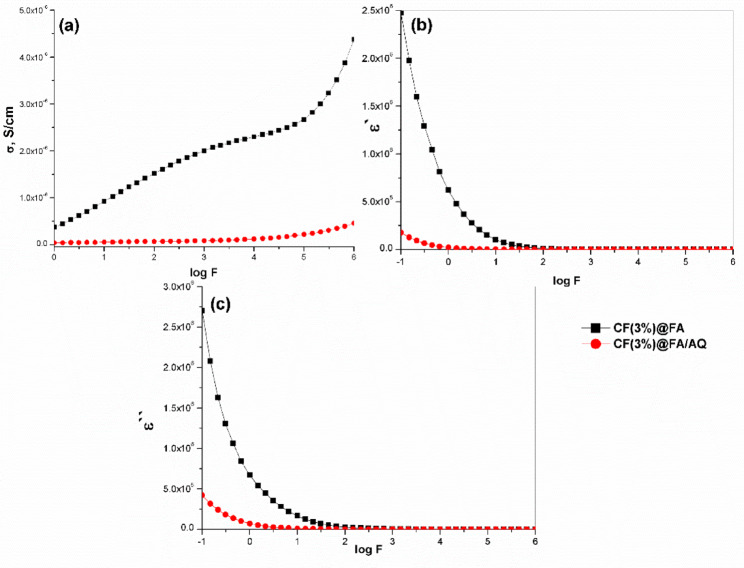
Comparative figure for electric and dielectric properties between CFs(3%)@FA/AQ and CFs(3%)@FA geopolymers with frequency variation

Based on the comparative results, Fig. 13 presents a differentiation between CFs(3%)@FA/AQ and CFs(3%)@FA geopolymers exhibiting the most favorable electric and dielectric properties. The incorporation of CFs significantly increased the electrical conductivity of CFs(3%)@FA (σ = 4.4 × 10^–6^ S/cm) at frequency 10^6^ Hz by six orders of magnitude compared to the CFs(3%)@FA/AQ geopolymer. The conductivity difference observed between CFs(3%)@FA and CFs(3%)@FA/AQ can be attributed to AQ, where non-polar [SiO_2_]n negatively impacted the flowability of the dispersed CFs. This outcome aligned with the findings reached by Wang et al. ^119^. Furthermore, the electrical conductivity of N-A-S–H gel can be increased by removing AQ, creating a more porous and less dense matrix with more free mobile ions. The observed variations in dielectric properties are likely caused by the rearrangement of charge carriers, dipoles, impurities, and lattice defects within the material. This redistribution leads to polarization effects, ultimately resulting in distinct dielectric relaxation behaviors ^120^.

#### Resistivity measurement

**Fig. 14:**
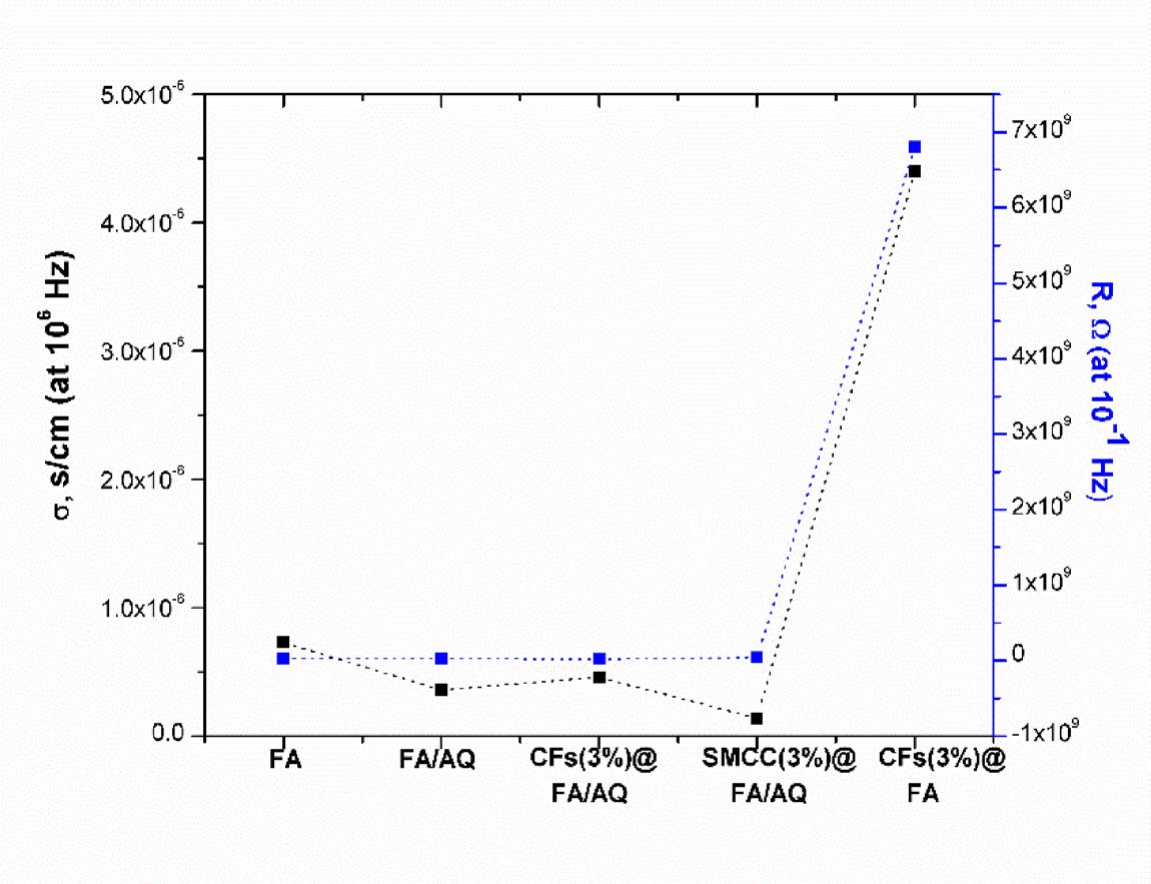
Variation in electrical conductivity (at 10^6^ Hz) and resistance (at 10^–1^ Hz) for geopolymer matrices with different fillers

The samples’ resistivity was determined by measuring their electrical resistance. Capacitance measurements revealed that high electrical resistivity was associated with low applied frequencies ^121^. Therefore, the variation in resistance and conductivity for the prepared FA-based geopolymers is presented in Fig. 14 applying the lowest (10^–1^ Hz) and the highest (10^6^ Hz) frequency values, respectively. From FA geopolymer to SMCC(3%)@FA/AQ geopolymer, there is a slight increase in resistance and a decrease in the conductivity values for SMCC(3%)@FA/AQ, attributed to the integration of AQ and SMCC. The incorporation of quartz possesses significant electrical resistivity, indicating that it effectively hinders the flow of electric current by interrupting conductive pathways in the original material ^122^.

The conductance reaches its higher point for the CFs(3%)@FA geopolymer due to its 0% AQ content. However, the resistance increases (6.8 × 10^9^ Ω) when the optimum frequency (10^–1^ Hz) condition is applied. The rise in resistance and conductivity of CFs(3%)@FA might stem from the influence of the applied frequency. Zhou et al. ^123^ identified three potential mechanisms that may govern the electrical resistivity change: the creation of conductive pathways via a percolation network of carbon fibers (CFs); the ion mobility within the polymer matrix; and a quantum tunneling percolation effect. These phenomena can be explained as follows: at low frequencies, conductivity is restricted by the requirement for direct physical contact or quantum tunneling between fibers. To traverse non-conductive gaps in this state, electrons must undergo an energy-intensive “hopping” process. Because of the tortuosity of the N-A-S–H matrix and interfacial polarization, these gaps act as resistive barriers. Consequently, the system is governed by a dominant ionic conduction regime, which is inherently slower since it depends on ion diffusion through the matrix or inter-fiber spaces, thereby increasing resistance. In high-frequency ranges, carbon fiber conductivity is primarily driven by improved percolation and inter-fiber network connectivity. The narrow gaps between filaments function as micro-capacitors, facilitating capacitive coupling. This effect allows electricity to bypass the bottlenecks present at lower frequencies by flowing as displacement current. As a result, the electrical response is dominated by electronic conduction regime, where the reduction in capacitive reactance at higher frequencies leads to significantly lower impedance and enhanced conductivity^124–127^.

Controlling the electrical conductivity of geopolymers is crucial for their application in piezoelectric and corrosion-resistant components. High resistivity correlates with slower corrosion rates, underscoring the importance of conductivity enhancement for diverse applications. Structured carbon-based additives offer a potential solution ^128,129^. Table 4 demonstrates comparable electric and dielectric properties of the prepared geopolymers across the surveyed literature. These findings highlight the suitability of these materials, with high electrical conductivity, for high-frequency photonic, ferroelectric, and electro-optic applications. The SMCC(3%)@FA/AQ demonstrates the least conductive properties and superior insulating properties compared to other geopolymers. In contrast, the CFs(3%)@FA geopolymer matrix exhibits higher values of electric and dielectric properties compared to other matrices ^129^. This renders it effective for electromagnetic interference shielding ^130^ and self-sensing concrete.

**Table 4:** Comparison of the electrical properties of our fabricated geopolymers with various recorded samples

Sample	σ (S/cm)	ε′	References
FA	7.3 × 10^–7^	2.25 × 10^5^	This study
FA/AQ	3.6 × 10^–7^	1.2 × 10^5^	This study
SMCC(3%)@FA/AQ	1.4 × 10^–7^	7 × 10^4^	This study
CFs(3%)@FA	4.4 × 10^–6^	2.5 × 10^6^	This study
CFs(3%)@FA/AQ	4.6 × 10^–7^	1.8 × 10^5^	This study
Natural zeolite-based geopolymer composites	(2–10) × 10^–6^	-	^128^
Electronic electroactive polymers	10^–9^—10^–6^	4.2—5.5	^120^
Fly Ash Geopolymer/Sugarcane Bagasse Composites	-	3.6 × 10^3^	^113^
Graphene-Reinforced Geopolymer	6.05 × 10^−14^	–	^131^
Fly ash KOH-based geopolymer	(3–9) × 10^–4^	(1—2.5) × 10^4^	^132^

## Conclusion

The main findings of this study can be summarized as follows:

- The study successfully developed multifunctional fly ash (FA)-based geopolymers using waste-derived additives, carbon fibers (CFs) and stabilized microcrystalline cellulose (SMCC). Carbon fibers (CFs) were successfully prepared by stabilization and carbonization processes for cotton precursor at elevated temperatures.
- The produced geopolymers were activated by a combination of NaOH-quartz (AQ) and sodium silicate solution (glass water) at ambient conditions.
- SEM micrographs have proved that incorporating NaOH-activated quartz (AQ) significantly densified the geopolymer matrix and reduced the natural porosity and voids found in neat FA geopolymer, restricting ion mobility.
- The addition of 3% SMCC promoted a dense, amorphous sodium aluminum silicate hydrate (N-A-S–H) network. This effective micro-void filling resulted in a peak compressive strength of 18.1 MPa.
- The integration of 3% CFs into the FA matrix, CFs(3%)@FA, achieved a six-order-of-magnitude increase in electrical conductivity by overcoming the effects of the quartz phase.
- While CFs enhanced conductive pathways, the SMCC(3%)@FA/AQ geopolymer demonstrated superior insulating properties, proving that these additive can tune the geopolymer for insulator roles.
- These multi-functional geopolymers are strong candidates for energy conversion applications, warranting further research into the “percolation threshold” of carbon-based additives.

## Data Availability

The authors declare that the data supporting the findings of this study are available within the paper file. Should any raw data files be needed in another format they are available from the corresponding author upon reasonable request.
